# Human Exposure to Herpesvirus B–Seropositive Macaques, Bali, Indonesia

**DOI:** 10.3201/eid0808.010467

**Published:** 2002-08

**Authors:** Gregory A. Engel, Lisa Jones-Engel, Michael A. Schillaci, Komang Gde Suaryana, Artha Putra, Agustin Fuentes, Richard Henkel

**Affiliations:** * University of New Mexico, Albuquerque, New Mexico, USA;; † Udayana University, Denpasar, Bali, Indonesia;; ‡ Central Washington University, Ellensberg, Washington, USA;; § Georgia State University, Atlanta, Georgia, USA

**Keywords:** herpesvirus B, primate, zoonoses, macaques, disease transmission, CeHV-1

## Abstract

Herpesvirus B *(Cercopithecine herpesvirus 1)* has been implicated as the cause of approximately 40 cases of meningoencephalitis affecting persons in direct or indirect contact with laboratory macaques. However, the threat of herpesvirus B in nonlaboratory settings worldwide remains to be addressed. We investigated the potential for exposure to herpesvirus B in workers at a “monkey forest” (a temple that has become a tourist attraction because of its monkeys) in Bali, Indonesia. In July 2000, 105 workers at the Sangeh Monkey Forest in Central Bali were surveyed about contact with macaques (*Macaca fascicularis*). Nearly half of those interviewed had either been bitten or scratched by a macaque. Prevalence of injury was higher in those who fed macaques. Serum from 31 of 38 Sangeh macaques contained antibodies to herpesvirus B. We conclude that workers coming into contact with macaques at the Sangeh Monkey Forest are at risk for exposure to herpesvirus B.

Herpesvirus B (*Cercopithecine herpesvirus 1* [CeHV-1]) is an alphaherpesvirus endemic to macaques of South and Southeast Asia (1). In macaques, the usual host, CeHV-1 causes mild symptoms, similar to the effects of herpes simplex virus 1 in humans (1). Clinical findings in macaques usually consist of oral or perioral vesicular lesions. After initial infection, CeHV-1 remains latent in the dorsal root or trigeminal ganglia of an infected macaque and can be shed periodically through herpetic lesions.

In contrast to its benign course in macaques, in humans CeHV-1 produces a fulminating meningoencephalitis with a mortality rate approaching 70% (2). Since first reported in the 1930s, a total of 43 cases of CeHV-1 have been diagnosed worldwide, all reported from the United States, Great Britain, or Canada, exclusively in people who had direct or indirect contact with laboratory macaques (2–6).

Several modes of primate-to-human transmission have been implicated, most involving direct exposure of tissue or fluid from an infected macaque. Weigler’s 1992 review of human CeHV-1 cases (1) found that most were infected through direct bite and scratch wounds: one case resulted from direct contamination of a preexisting wound with monkey saliva, two cases occurred after lacerations from culture bottles containing macaque cells, and two occurred in persons punctured by needles previously used in macaques. One case of human-to-human transmission has been documented, when infection developed in the wife of a man who subsequently died of a CeHV-1 infection. She had a rash on her finger that came into contact with a vesicular lesion on her husband’s arm, at the site of a monkey bite. The most recent documented case occurred in 1997 at the U.S. Yerkes Regional Primate Center, where a young worker who received an ocular exposure with contaminated body fluids from a CeHV-1–positive macaque became ill and subsequently died (6).

Two published case series have studied transmission of CeHV-1 from primates to humans. Friefeld et al. (7) examined prevalence of antibodies to CeHV-1 in primate handlers exposed to bites, scratches, needle-sticks, and mucosal splashes from laboratory macaques. None of the 166 exposed persons had antibodies to the virus. Similarly, in a small study of eight persons bitten by pet macaques, none seroconverted (8). Nevertheless, the threat of herpesvirus B has led the Centers for Disease Control and Prevention to recommend strict precautions for persons who come into contact with monkeys in occupational settings (2,6,9,10).

The threat of CeHV-1 to humans in nonlaboratory contexts worldwide has yet to be studied, despite the fact that the laboratory macaques that harbor the virus originated in Asia or are descendents of macaques originating there. Macaque species range throughout South and Southeast Asia and have adapted well to human-altered environments. In turn, macaques have become incorporated into religious mythology and local culture. Hindus in Indonesia, Nepal, and India, for example, regard macaques as sacred (11), and in many areas protected macaque populations have thrived alongside dense human settlements for centuries. On the Indonesian island of Bali, more than 44 Balinese Hindu temples have, over the centuries, become refuges for populations of free-ranging macaques (11). These monkeys subsist at least in part on the food and flower offerings left by Balinese Hindu worshipers. Over time, some of these temples have become tourist destinations known as “monkey forests,” where macaques are the premier attraction. At the Sangeh Monkey Forest in Central Bali, dozens of local photographers make a living by enticing macaques to climb onto visitors and selling the photos of visitors posing with macaques. However, photographers and tourists are by no means the only humans who come into contact with these macaques. At Sangeh, three troops of macaques, a total of >200 monkeys, range throughout the monkey forest, along a road lined with merchants’ shops, and into the adjacent town. Their daily travels afford ample opportunity for contact with shop owners and others who pass near the monkey forest.

This study investigated human-macaque contact in people who work in and around the Sangeh Monkey Forest. Our aim was to examine the risk of exposure to CeHV-1 from Sangeh’s macaques. We addressed two principal questions: 1) does human-primate contact capable of transmitting CeHV-1 occur in this context? and 2) are the Sangeh macaques seropositive for antibodies to this virus?

## Methods

### Study Site

The Sangeh Monkey Forest is located in the Abiensemal District, central Bali, approximately 20 km north of Denpasar, Bali’s most populous city. Built in the 17th century by the royal family of Mengwi, it is now maintained by the people of the village of Sangeh. The macaque (*Macaca fascicularis*) population at Sangeh totals approximately 200 animals. Their range extends through the monkey forest proper and across a main road that abuts the area. The monkey forest, a 6-hectare stand of *Dipterocarpus hasseltii* and other climax forest trees with heights up to 40 m, actually provides little food for the macaques. Most of their caloric intake is from daily provisions provided by temple workers and food given to them by visitors.

Visitors interact with macaques mainly in an open area adjacent to the principal temple structures. The main entrance to the monkey forest is reached by a promenade lined by shops offering clothing and souvenirs, in addition to peanuts and bananas for the macaques. Groups of macaques also make their way down this thoroughfare or along the shop roofs during their daily ranging.

### Ethnographic Surveys

The local temple committee provided researchers with a list of 250 persons whose work in and around the monkey forest brought them into regular proximity with the macaques. Most persons were either photographers or merchants whose shops lined the road leading to the monkey forest. Of this group, 105 persons (42%) volunteered to participate in the study. In July 2000, a questionnaire that focused on human-primate contact, written in Bahasa Indonesia, the national language of Indonesia, was administered by Balinese team members. Information requested included the type and number of contacts with Sangeh’s macaques, injuries or sequelae resulting from macaque bites and scratches, and treatments of those injuries.

### Field Protocol

Universal precautions were observed during animal handling and specimen collection to minimize the risk of pathogen transmission between researchers and nonhuman primate subjects. All methods were reviewed and approved by the University of New Mexico’s Institutional Animal Care and Use Committee.

Macaques were opportunistically darted within the monkey temple area and surrounding forest by using a Pneu-Dart air-powered pistol (Pneu-Dart Inc., Williamsport, PA). Darts were loaded with 15 mg of Telazol (Fort Dodge Laboratories, Fort Dodge, IA; tiletamine HCl/zolazepam HCl) to ensure initial sedation. Immediately after darting, the macaque was moved to a secluded area and <5 mg/kg of supplemental Telazol was administered for sedation. Six milliliters of blood was withdrawn from the femoral vein, placed in a serum separator tube, and centrifuged in the field to extract the serum. Sera were frozen and stored at –20°C. Dental eruption sequence was recorded and used as a proxy measure of chronologic age. Macaques were observed and allowed to recover from anesthesia in a quiet area before being released. No macaques were injured as a result of this protocol.

### Laboratory Techniques and Data Analysis

Enzyme-linked immunosorbent assays (ELISA) to detect antibodies to CeHV-1 in macaque sera were performed at the B-Virus Reference Laboratory at Georgia State University (12). Questionnaire and serologic data were entered into a spreadsheet, and univariate analysis was performed with the JUMP-IN 4 statistical software package (SAS Institute, Inc., Cary, NC, version 4). The association between macaque CeHV-1 seropositivity and age was determined by chi-square test. Prevalence ratios, regarded by some as the most appropriate tool for analyzing cross-sectional studies, were calculated to describe associations between demographic variable and feeding behavior and prevalence of bite and scratch exposure (13,14). For all variables the category with the lowest prevalence was used as the referent. Calculation of prevalence ratios and 95% confidence intervals [CI] was performed with the NCSS Statistical Software package (Kaysville, UT).

## Results

### Seroprevalence of Antibodies to CeHV-1 in Macaques

Demographic and serologic data on the macaques sampled are shown (Table 1).

**Table 1:** Seroprevalence of antibodies to *Cercopithecine herpesvirus 1* (herpesvirus B) in Sangeh macaques (*Macaca fascicularis*), Bali

Age group^a^/sex	No. ELISA positive/total (%)
Juvenile	
Male	1/4 (25)
Female	—
Total	1/4 (25)
Subadult	
Male	1/4 (25)
Female	1/2 (50)
Total	2/6 (33.3)
Adult	
Male	21/21 (100)
Female	7/7 (100)
Total	28/28 (100)
All ages	
Male	23/29 (79.3)
Female	8/9 (88.8)
Total	31/38 (81.6)

^a^Juveniles are defined as 1–3 years of age; subadults as 3–5 years of age; adults as >5 years of age. —, no data; ELISA, enzyme-linked immunosorbent assay.

Thirty-one (81.6%) of the 38 sampled macaques tested positive for antibodies to CeHV-1. One (25%) of the four juveniles, two (33.3%) of the six subadults, and all 28 adults (100%) were seropositive. The association of increased seroprevalence with increasing age was statistically significant (p<0.0001, chi square). These figures are consistent with those of other seroprevalence studies performed on captive and noncaptive macaques (15–18).

### Demographics of the Human Study Population

Demographic data for the human study participants are summarized in Table 2. The mean age of the study population was 35.6 years (standard deviation 12.0). The median age was 35 years (range 18–75); 63.8% of respondents were male. The three most common occupations were merchants (34.3%), photographers (24.8%), and farmers (18.1%). Consistent with cultural norms, 35 of the 36 merchants were women who owned shops along the main promenade leading to the temple area. Of the 38 female respondents, all but 3 were merchants. All respondents were Balinese Hindus residing in the village of Sangeh.

**Table 2:** Demographic characteristics of human study participants, Sangeh, Bali

Demographic characteristic	No. (% of total)
All persons	105 (100)
Age group	
<20	11(10.5)
20–29	27 (25.7)
30–39	26 (24.8)
40–49	31 (29.5)
>49	10 (9.5)
Sex	
Male	67 (63.8)
Female	38 (36.2)
Marital status	
Single	29 (27.6)
Married	75 (71.4)
Widowed	1 (1)
Education	
<6th grade	38 (36.2)
7th–9th grade	23 (21.9)
10th–12th grade	38 (36.2)
University	6 (3.8)
Occupation	
Merchant	36 (34.3)
Photographer	26 (24.8)
Farmer	19 (18.1)
Security	7 (6.7)
Government employee	5 (4.8)
Traditional guard	5 (4.8)
Laborer	4 (3.8)
Other	3 (2.9)

### Bite and Scratch Results

Prevalences of bite and scratch injuries are summarized in Table 3. Results from the survey showed that 76.9% of persons had touched or been touched by a macaque. Almost a third (29.5%) reported that they had been bitten on at least one occasion by a macaque; 39% had been scratched at least once; some persons reported being both bitten and scratched. Of the 51 injured, 94.1% were holding food at the time of the injury.

**Table 3:** Prevalence of bite and scratch injuries from monkeys to humans, Sangeh, Bali

Descriptor	No. (%) of persons (n = 105)
Bitten	31 (29.5)
Bitten more than once	7 (6.7)
Scratched	41 (39.0)
Scratched more than once	15 (14.3)
Bitten or scratched	51 (48.6)
Possessed food at time of injury	48 (94.1)
All persons	105 (100)

### Anatomic Distribution of Injuries, Sequelae, and Wound Treatment

By anatomic distribution, 64.7% of the 51 injuries were to the hands, 7.8% to the arms, 7.8% to the legs, 11.8% to the head, and 7.8% to the back or buttocks. Of the 51 persons bitten or scratched, 51% reported that the wound had bled, 9.8% reported a rash at the bite site, 11.8% noted fever after their injury, 3.9% had headache, and 5.9% had generalized weakness. None of those interviewed reported symptoms consistent with previously described cases of CeHV-1 infection.

In terms of wound treatment, 54.9% of those injured did not use any kind of treatment for their scratch or bite injuries, 21.6% washed the wound with an antiseptic solution, and 19.6% washed with soap. Of the injured, 11.7% were treated at a medical clinic, and 2% were treated by an herbalist. Five of the persons treated at the medical clinic received antibiotics; none received antiviral prophylaxis.

### Prevalence Ratios for Exposures to Monkey Bites and Scratches

Table 4 presents prevalence and prevalence ratios for exposure to monkey bites or scratches, by respondent’s age group, sex, occupation, level of education, and whether the respondent fed macaques. Persons in their twenties had the highest prevalence of exposure (63%) while those <20 years of age had the lowest (9%). The exposure rate for male participants (62.7%) was higher than that for females. The prevalence ratio for males was 2.6, indicating that their exposure was more than two and a half times as common as that of female study participants. Of occupations represented by more than five persons, farmers (94.7%) had the highest exposure prevalence, followed by photographers (57.7%), merchants (25%), and security guards (14.3%). For farmers, the prevalence ratio was 6.6, with a 95% CI (1.61, 78.46). Exposure rates were higher in persons with grade school or less education (57.9%) and middle school education (56.5%) than those who had reached high school (39.5%) and university (16.7%).

**Table 4:** Prevalence and prevalence ratios for exposure to monkey bites or scratches by different variables, Sangeh, Bali

Variable	Total no. (%) persons exposed	Prevalence ratio	95% CI	p value
Age group (yrs)				
<20	11 (9.0)	1.0	—	—
20–29	27 (63.0)	6.9	1.75, 117.30	0.045
30–39	26 (50.0)	5.5	1.34, 93.86	0.080
40–49	31 (45.2)	5.0	1.21, 84.81	0.100
>49	10 (60.0)	6.0	1.47, 113.93	0.056
Sex				
Female	38 (23.7)	1.0	—	—
Male	67 (62.7)	2.6	1.55, 5.26	0.002
Occupation				
Security	7 (14.3)	1.0	—	—
Merchant	36 (25.0)	1.7	0.41, 29.67	0.564
Photographer	26 (57.7)	4.0	1.08, 66.80	0.138
Farmer	19 (94.7)	6.6	1.90, 108.43	0.041
Other	17 (47.1)	2.2	0.80, 55.40	0.215
Education				
University	6 (16.7)	1.0	—	—
High school	38 (39.5)	2.4	0.64, 38.91	0.356
Middle school	23 (56.5)	3.4	0.93, 55.53	0.190
Grade school or none	38 (57.9)	3.5	0.99, 56.51	0.177
Fed monkeys				
No	15 (6.7)	1.0	—	—
Yes	90 (55.6)	8.3	2.08, 142.05	0.029

CI, confidence intervals.

Most men (89.4%) and women (79%) reported that they had offered food to macaques. Injury was more prevalent in persons who reported feeding macaques (55.6%) than in those who denied feeding them (7.1%). The prevalence ratio in those who fed monkeys was 8.3, indicating that exposure in those who fed monkeys was nearly eight times more common than in those who did not feed monkeys.

## Discussion

### Exposure to Macaque Bites and Scratches at the Sangeh Monkey Forest

The survey data presented in this study suggest that many workers in and around the Sangeh Monkey Forest have been bitten or scratched by a macaque. Serologic data show that >80% of these macaques have been exposed to CeHV-1. Current understanding of the pathophysiology of this virus predicts that seropositive animals periodically shed it through mucosal lesions (1,17–19). Therefore, these workers report injuries that put them at risk for exposure to the virus.

### Wound Care and the Risk for Pathogen Transmission

In contrast with laboratory settings, where protocols regarding care of nonhuman primate-inflicted wounds specify immediate and thorough decontamination, awareness of the risk of zoonotic disease in workers at Sangeh is low. Data on care of macaque bite and scratch wounds reflected this. Lack of prompt and rigorous wound care may thus pose added risk for transmission of CeHV-1 and other nonhuman primate-borne pathogens for workers and visitors at Sangeh.

### CeHV-1 a Cause of Human Disease on Bali?

The above data suggest that human-macaque contact capable of transmitting CeHV-1 is relatively common at Sangeh. Indeed, these data may represent only a small fraction of the human-macaque contact occurring there. Wheatley (11) reported that up to 40% of visitors to Sangeh are bitten by a macaque. Given that thousands of tourists visit Sangeh during a typical month, a reasonable estimate of the annual number of injuries inflicted by macaques is in the thousands, and Sangeh is but one of a handful of monkey forests on Bali that draw large numbers of visitors. Yet no case of human CeHV-1 infection has been reported in Bali, either in association with monkey forests or in any other nonlaboratory context (K. Suaryana, pers. comm.). Several explanations for this observation can be offered.

Recent work supports the existence of three genotypes of CeHV-1, each associated with a distinct species of macaque (20). It has been suggested that only the strain associated with rhesus macaques (*M. mulatta*), the most commonly used laboratory macaque, causes virulent disease in humans (20). This hypothesis is based on the observation that, when the identity of the source animal was known, human CeHV-1 infection was associated with exposure to rhesus macaques but never solely with exposure to other macaque species, including *M. fascicularis*, the species found in Bali, and the second-most commonly used macaque in laboratory research.

The hypothesis that only certain species of macaques may carry a pathogenic strain of CeHV-1 has not been tested. Almost all such infections have occurred in laboratory settings where rhesus macaques constitute most of laboratory nonhuman primates. In addition, rhesus macaques tend to be the more aggressive species. Thus, rhesus macaques may simply cause more injuries and hence be associated with more viral exposures than other macaque species. Furthermore, no case of CeHV-1 infection in humans has been reported in India or Nepal, countries where human-macaque contact is known to occur outside the laboratory and where the predominant macaque species is the rhesus monkey. However, no active surveillance for CeHV-1 is carried out in Nepal and India, and persons diagnosed with encephalitis in these countries are highly unlikely to be tested for this virus.

Another possible explanation for the lack of reported human CeHV-1 cases is that opportunities for exposure to actual virus may be rare. Previous research suggests that, in the laboratory setting, macaques seropositive for CeHV-1 antibodies, even under certain kinds of stress, infrequently shed the virus (17,19). If the macaques at Sangeh behave similarly and shed the virus infrequently, the opportunity for exposure may be rare. One must also take into account the probability that the macaques that bite or scratch carry CeHV-1. Specifically, infant and juvenile macaques are less likely to test positive for anti-CeHV-1 antibodies than older macaques and are thus, as a group, probably less likely to shed virus (1,15). Data from the Ubud Monkey Forest (A. Fuentes, pers. comm.) suggest that adult male and female macaques accounted for approximately half of all bites, with the remainder attributed to juveniles. At least half the bites, therefore, are caused by macaques that are less likely to harbor the virus. Unfortunately, no large-scale studies of CeHV-1 shedding in wild macaques have been performed to date, so the rate at which these animals shed the virus is unknown.

CeHV-1 infection in humans might also be underreported if the disease is rare, especially since awareness of this virus among health-care providers in Bali is low. The symptoms of infection might be mistaken for those of other neurologic diseases, such as polio or Guillain-Barré syndrome. Thus it is theoretically possible that humans reporting mild sequelae following monkey bites and scratches might be describing a mild variant of B virus syndrome. We are aware of no research examining the prevalence of antibodies to CeHV-1 in persons with neurologic syndromes in Asia.

Finally, resistance to CeHV-1 in the exposed human population may explain the lack of reported cases. Human populations living in proximity to the monkey forests in Bali have been living commensally with macaques for centuries. These populations could conceivably acquire immunologic resistance to the virus as a result of frequent exposure over time.

More data are needed to assess whether CeHV-1 poses a substantial public health threat to workers at Sangeh. Serologic data from humans who have been scratched or bitten by macaques could help to determine whether these persons have been exposed to the virus. In addition, a thorough search for cases of human CeHV-1 infection, in Southeast Asia as well as in South Asia, could yield further insight into the epidemiology of this virus in the human population. However, the virus has not surfaced as a recognized infectious threat for humans in areas where the two species have lived commensally for centuries.

### Public Health Significance of Nonhuman Primate Zoonoses

Data such as those presented in this study can play an important role in preventing the emergence of primate zoonoses. By understanding where and how interspecies contact occurs we may take informed steps toward reducing the likelihood of interspecies pathogen transmission. Specifically, information about interspecies pathogen transmission may help to identify priority areas for intervention to reduce the emergence of nonhuman primate-borne zoonoses.

### Implications and Recommendations for Public Health Practice

Over the past decades, the widespread use of laboratory nonhuman primates as models for the study of human diseases has led to the exposure of laboratory workers to infectious agents endemic in nonhuman primates. Relatively little is known about the epizootology of infectious agents harbored by nonlaboratory macaques and even less is known about the transmission of these agents from macaques to humans with whom they come into contact. CeHV-1 is one of a few infectious agents known to be transmitted from macaques to humans. Serologic, virologic, and molecular studies have demonstrated that a handful of laboratory workers have acquired infection with *Simian foamy virus* as well as *Simian immunodeficiency virus* and simian retrovirus, though no known adverse health effects were associated with these infections (21–29). Very little is known about the effects of these viruses on humans, since the number of seroconverters is low, <10 cases for each virus. No serologic studies outside laboratory settings have been conducted. Given the paucity of data on the effects of CeHV-1 and other endemic macaque pathogens on humans, especially in nonlaboratory settings, reducing the kinds of interspecies contact most likely to lead to pathogen transmission would be prudent. Our data suggest some possible interventions. Because workers who fed macaques were far more likely to be bitten or scratched than their other colleagues, an intervention aimed at reducing injuries in workers might logically focus on feeding practices. Restricting feeding to specially trained personnel who distribute food to macaques in such a manner as to avoid physical contact with them is one strategy that has worked effectively at other monkey forests (A. Fuentes, pers. comm.). Also, since approximately two thirds of those who are injured report injuries to the hands, the use of protective gloves should be advocated for personnel coming into frequent contact with monkeys. Of course, implementing these kinds of changes would require a commitment from the community to change the way the monkey forests operate.

Another incentive for enacting such changes is that monkey forests and the macaques that live in them are valuable cultural and economic resources to the communities in which they are located. Disease transmission in the opposite direction, namely human to nonhuman primate, may threaten these macaques. If so, regulating interspecies contact could help to preserve the monkey forests as an economic resource for the community. Perhaps a long-term strategy to preserve monkey forests will recognize the importance of minimizing infectious risks to both humans and macaques.
